# SERS-Driven Evolution of Lateral and Vertical Flow Assays in Medical Diagnostics

**DOI:** 10.3390/bios15090573

**Published:** 2025-09-01

**Authors:** Boyou Heo, Ho Sang Jung

**Affiliations:** School of Biomedical Engineering, Korea University, Seoul 02481, Republic of Korea; heo8760@korea.ac.kr

**Keywords:** lateral flow assay, vertical flow assay, plasmonics, diagnostics, on-site detection

## Abstract

Surface-enhanced Raman scattering (SERS) has emerged as a powerful signal amplification strategy to address the inherent limitations of conventional flow-based diagnostic methods such as lateral flow analysis (LFA) and vertical flow analysis (VFA). By incorporating SERS-active nanostructures into these platforms, SERS-integrated LFA and VFA systems have significantly improved sensitivity, specificity, and multiplexing performance while maintaining the simplicity and portability of conventional approaches. In this review, we summarize recent advances in SERS-enhanced flow-based diagnostics with a focus on exogenous and endogenous disease detection. Exogenous targets include viral antigens, bacterial pathogens, and foodborne contaminants such as mycotoxins and antibiotic residues. Endogenous applications include therapeutic drug monitoring, inflammation profiling, cancer biomarker detection, and exosome-based molecular subtyping. We highlight the structural differences between LFA and VFA approaches and their impact on analytical performance, and explore the advantages of SERS-integrated platforms for rapid and multiplexed detection in complex biological matrices. Finally, we provide an overview of key technical challenges, such as signal reproducibility, matrix interference, and device integration, and discuss future directions for clinical implementation of SERS-based flow diagnostics in point-of-care settings.

## 1. Introduction

The COVID-19 pandemic, which had resulted in over 770 million confirmed cases and nearly 7 million deaths globally by the end of 2024, underscored the critical need for rapid, decentralized, and accessible diagnostic tools [1]. Beyond COVID-19, the post-pandemic era has witnessed the emergence and re-emergence of several infectious diseases, highlighting the persistent threat of global health crises. For instance, in 2022, the World Health Organization declared an international health emergency due to the unprecedented spread of mpox (formerly known as monkeypox), which affected numerous countries outside of its traditional endemic regions [2]. In 2024, a significant outbreak of Oropouche fever occurred in Latin America, with over 10,000 confirmed cases reported across countries like Brazil, Peru, and Colombia [3]. Additionally, the highly pathogenic avian influenza H5N1 has expanded its host range, infecting over 48 mammalian species and causing substantial wildlife mortality, raising concerns about potential human transmission [4]. These events illustrate that the emergence of infectious diseases is an ongoing challenge, necessitating the development and deployment of efficient diagnostic platforms. Point-of-care (PoC) diagnostic devices, such as lateral flow assays (LFA) and vertical flow assays (VFA), have proven invaluable in facilitating rapid and decentralized testing during such outbreaks [5]. Their ease of use, cost-effectiveness, and ability to deliver prompt results make them essential tools in the early detection and containment of infectious diseases [6]. Among PoC technologies, LFA is one of the most widely adopted formats and is familiar to the public through home pregnancy tests and rapid COVID-19 antigen kits [7]. LFA operates on the principle of capillary-driven flow across a porous membrane. Typically, a liquid sample is applied to a sample pad, then it migrates through a conjugate pad containing nanoparticle-labeled antibodies, and flows laterally along a nitrocellulose membrane. When a target analyte is present, a sandwich complex forms at the test line, producing a visible signal, often through colorimetric change [8,9]. In contrast, VFA is designed with a vertical flow pathway, where the sample moves perpendicularly through stacked membranes or porous layers [10]. This configuration enables rapid filtration, minimal lateral diffusion, and reduced assay time [11]. Unlike LFA, which relies solely on capillary action, VFA can harness additional driving forces such as gravity and externally applied pressure, which is often generated by a syringe or vacuum system, to accelerate fluid movement [12]. As a result, VFA often achieves higher sensitivity and multiplexing capability than LFA due to its capacity for spatial reagent separation, lower background interference, and compatibility with advanced signal readouts [13].

However, both LFA and VFA platforms face intrinsic challenges, especially in terms of sensitivity, quantification, and multiplexing capacity. These limitations become important when detecting low concentration biomarkers, such as those associated with exogenous or endogenous diseases [14]. In order to address the aforementioned issues, recent innovations have incorporated surface-enhanced Raman scattering (SERS) technology into flow-based assay platforms [15,16]. SERS offers ultra-sensitive molecular detection capabilities by amplifying Raman signals of reporter molecules using metallic nanostructures, thereby bridging the sensitivity gap while retaining the portability and simplicity of flow assays [17]. SERS is a powerful vibrational spectroscopy technique that dramatically enhances the Raman signals of molecules adsorbed on or near metal nanoparticles, typically composed of gold or silver. This enhancement arises from two primary mechanisms: the electromagnetic effect, which amplifies the local electric field due to surface plasmon resonance, and the chemical effect, which involves charge transfer interactions between the analyte and the metal surface [18]. Collectively, these mechanisms empower SERS to attain single-molecule sensitivity, high specificity, and multiplexing capabilities [19,20,21]. Their ability to perform multiplexed, quantitative analysis from minimally invasive samples makes them ideal for longitudinal monitoring of disease progression and therapeutic response [22]. The convergence of SERS technology with flow-based diagnostic platforms thus represents a transformative advancement in PoC testing, particularly in resource-limited environments and during global health emergencies. By enabling rapid, accurate, and information-rich detection of disease biomarkers, SERS-integrated LFA and VFA systems hold immense potential to improve clinical decision-making, facilitate personalized treatment strategies, and strengthen public health surveillance.

In this review, we summarize recent advances in SERS-integrated lateral and vertical flow spectrometry, focusing on their architecture design, analytical strategies, and clinical applications. Exogenous targets refer to pathogen-derived analytes and external toxins, including viral antigens such as nucleocapsid or spike proteins, bacterial antigens or whole-cell markers, and microbial or foodborne toxins. Endogenous targets denote host-derived biomarkers that vary with disease state, including circulating proteins and peptides such as cTnI, CK MB, myoglobin, CRP, IL 6, SAA, PCT, and TSH, extracellular vesicles and exosomes bearing markers such as HER2, CEA, and MUC1, nucleic acids such as circulating microRNAs, and small molecule analytes relevant to therapy or metabolism such as flucytosine. Unless stated otherwise, biomarker refers to both antigen and antibody formats used in immunoassays. Representative case studies for these categories are presented in Section 3.1, Section 3.2, Section 3.3 and Section 3.4. Based on recent case studies in the field of exogenous and endogenous disease diagnosis, we discuss how these platforms have been designed to overcome existing limitations in sensitivity, quantification, and multiplexing. Based on these advances, we highlight current challenges and suggest future directions for the development of SERS-based PoC diagnostic systems.

## 2. Conventional Flow-Based Assays: LFA and VFA

**Figure 1:**
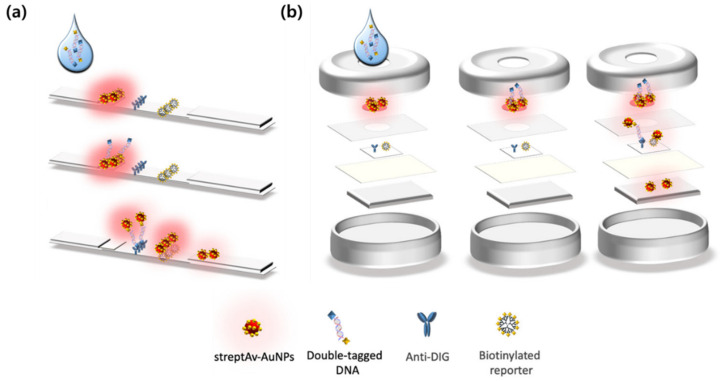
Representative Designs and Working Principles of LFA and VFA. Schematic illustration of (**a**) LFA and (**b**) VFA using strepAv-AuNPs for target detection. Reprinted (adapted) with permission from ref [23]. Copyright 2021 MDPI.

LFA and VFA have been widely adopted as diagnostic platforms in PoC settings due to their simplicity, low cost, and ability to provide rapid results without the need for complex instrumentation [23]. Both platforms rely on porous membranes to facilitate fluid transport, enabling the detection of biomarkers directly from biological samples. LFA primarily utilizes capillary action to drive lateral fluid flow, whereas VFA combines capillary forces with gravity or externally applied pressure to accelerate vertical flow through stacked membrane layers, thereby improving assay speed and efficiency [24,25]. As illustrated in Figure 1a, LFA typically consists of a series of horizontally aligned components, including a sample pad, a conjugate pad, a reaction membrane with test and control lines, and an absorbent pad. When a sample is applied, it is transported laterally through the device via capillary action. During this flow, the sample interacts with labeled detection probes, and target analytes are captured at the test line by immobilized recognition elements, generating a visible signal. The control line functions as an internal quality control to verify proper fluid migration. Figure 1b shows a typical configuration of VFA, which is structured in a vertically stacked format. A standard VFA device includes a sample application zone, a conjugation layer, a capture membrane, a support layer, and an absorbent pad. After the sample introduction, the fluid flows downward under the influence of gravity or applied pressure, while target analytes bind to capture agents embedded in the membrane. The signal is then generated on the same plane, and excess fluid is absorbed at the bottom to maintain continuous flow. This format supports rapid filtration, minimizes background interference, and enables efficient multiplex detection through spatial separation of capture zones. Although the two platforms differ in their structural orientation and flow mechanics, they operate on the same fundamental principle: target analytes bind to labeled detection probes during fluid migration and are subsequently immobilized by specific capture elements to generate a detectable signal. This shared mechanism, combined with ease of use and compatibility with low-resource settings, has led to their widespread application in pregnancy testing, infectious disease screening, and food safety monitoring [26,27,28]. Despite their practical advantages, LFA and VFA platforms face inherent limitations when applied to diagnostic applications that require high sensitivity, quantitation, and multiplexing. These challenges arise from the physical properties of porous membrane flow, which limit the analyte–probe interaction time and reduce the efficiency of molecular binding reactions, especially at low analyte concentrations. Furthermore, conventional visual readouts based on colorimetry have limited signal resolution and are difficult to quantify, especially in complex biological samples [5,7,13]. To overcome these structural and functional limitations, recent efforts have focused on integrating signal amplification strategies into flow-based platforms, with SERS emerging as a promising approach [29]. In strip-based PoC implementations, analytical enhancement factors for on-strip measurements typically range from 10^4^ to 10^6^ and are influenced by the nanotag structure, reporter loading, and acquisition conditions [30]. Gap-engineered Raman tags that confine the field within nanogaps often show single particle enhancement factors of approximately 10^8^ to 10^9^ under microscopic excitation, and when applied to lateral or vertical flow configurations they have yielded device-level limits of detection that are 10 to 20-fold lower than colorimetric readouts [31,32]. SERS-enabled nanoprobes functionalized with Raman reporters and biorecognition elements enable highly specific spectral detection without altering the basic analyte architecture [33,34]. This integration improves detection limits and signal resolution while maintaining the simplicity, portability, and fast analysis times of conventional LFA and VFA systems. SERS is structurally compatible with both horizontal and vertical configurations, facilitating the development of advanced platforms that allow for multiplexing and quantitative detection of samples with minimal processing [35,36].

In the following section, we examine recent advances in SERS-integrated LFA and VFA systems and explore how these hybrid technologies improve sensitivity, enable quantitative analysis, and expand multiplexing capacity to meet the growing demands of PoC diagnostics.

## 3. Recent Advances in SERS-Integrated LFA and VFA for Disease Diagnostics

### 3.1. SERS-LFA: Exogenous Diagnostic Case

**Figure 2:**
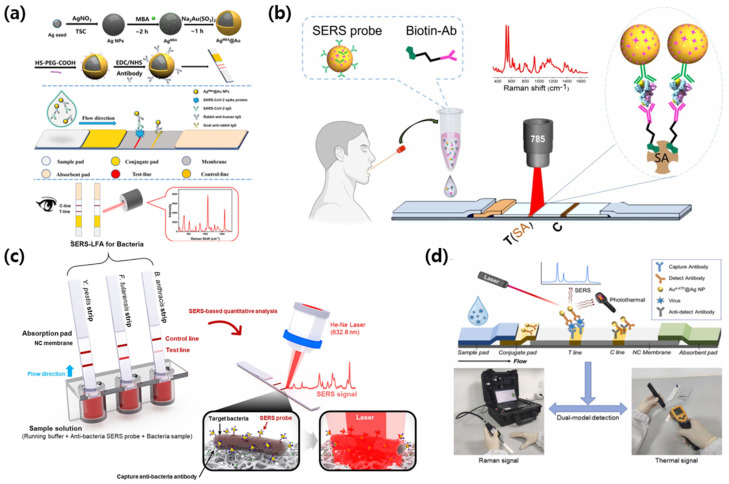
Representative SERS-integrated LFA platforms for exogenous disease diagnostics. (**a**) A dual-mode LFA platform using Ag@MBA@Au nanoprobes for colorimetric and SERS-based detection of SARS-CoV-2 IgG. Reprinted (adapted) with permission from ref [37]. Copyright 2022 ACS Publications. (**b**) Resonance SERS-LFA using IR808-labeled nanoprobes for highly sensitive monkeypox virus detection. Reprinted (adapted) with permission from ref [40]. Copyright 2025 Elsevier. (**c**) SERS-based LFA strips for multiplexed detection of high-risk bacterial pathogens. Reprinted (adapted) with permission from ref [44]. Copyright 2018 Elsevier. (**d**) Dual-modal SERS/photothermal LFA enabling sensitive and simultaneous antigen detection of respiratory viruses. Reprinted (adapted) with permission from ref [49]. Copyright 2023 Elsevier.

The exogenous diagnostic case refers to assays that target pathogen-derived analytes or external toxins. SERS-integrated lateral flow assays replace colorimetric reporters with Raman-encoded nanotags and enable sensitive, quantitative, and multiplex detection in complex matrices. The examples that follow are organized around four recurring designs: serology assays that measure virus-specific antibodies, antigen sandwich assays for viral antigens, pathogen-specific strip layouts for bacteria, and dual modal SERS/photothermal implementations that provide confirmatory readouts. Figure 2 summarizes recent representative applications of SERS-LFA platforms in the diagnosis of viral and bacterial diseases. For clarity in what follows, the exogenous diagnostic case denotes assays that target pathogen-derived analytes or external toxins, whereas the endogenous diagnostic case denotes assays that target host-derived biomarkers that vary with disease state.

Liang et al. [37] developed a dual-mode SERS-based LFA platform for the detection of SARS-CoV-2 IgG antibodies. Accurate and rapid detection of SARS-CoV-2-specific antibodies is essential for immune response monitoring and cohort-level surveillance [38]. Conventional ELISA offers high specificity and quantitation but is slow and equipment-dependent [39]. In contrast, colloidal gold-based LFA is rapid and simple but has limitations in sensitivity and qualitative analysis [7]. These limitations have prompted the development of hybrid diagnostic platforms that combine the operational simplicity of LFAs with the analytical performance of laboratory-based techniques. The system utilizes Ag nanoparticles coated with an ultrathin (~2 nm) gold shell, embedded with 4-MBA (4-mercaptobenzoic acid) as a Raman reporter (Ag@MBA@Au). This core–shell structure enhances Raman signal generation due to the strong electromagnetic field formed within the nanogap between the Ag core and the Au shell, while also offering the biocompatibility and surface functionalization benefits of gold. The nanoparticles were conjugated with anti-human IgG antibodies and incorporated into a lateral flow strip, where SARS-CoV-2 spike protein was immobilized on the test line. As shown in Figure 2a, the presence of SARS-CoV-2 IgG leads to a sandwich complex formation on the test line, resulting in a visible red band and a strong SERS signal. The Raman intensity was measured at 1075 cm^−1^, corresponding to the characteristic peak of 4-MBA. The assay achieved a limit of detection of 0.52 pg/mL in serum, which is approximately four orders of magnitude more sensitive than that of traditional colloidal gold-based LFAs. Clinical evaluation of 107 serum samples yielded results that closely matched those of ELISA, while outperforming commercial gold strip tests in both sensitivity and classification accuracy. The intra-assay and inter-assay coefficients of variation were 9.1% and 13.2%, respectively, indicating good reproducibility. These results support the strong potential of SERS-LFA platforms for serological diagnosis of COVID-19 in point-of-care settings.

Li et al. [40] introduced a resonance-enhanced SERS probe, based on the IR808 molecule for the detection of monkeypox virus (MPXV), to increase sensitivity and reduce background interference in virus detection. The re-emergence of MPXV highlights the need for highly sensitive, rapid and suitable diagnostic tools for early detection in field settings [41]. PCR remains the reference test, but its infrastructure requirements are impractical for use in PoC diagnostics [42]. Conventional antigen tests, on the other hand, are fast but insensitive, especially for low viral concentrations or complex specimen matrices such as pharyngeal swabs [43]. As a way to improve this, they used gold nanoparticles labeled with IR808, a near-infrared dye that exhibits strong Raman scattering when excited by a 785 nm laser. These nanoparticles are conjugated to a primary antibody targeting the MPXV antigen and serve as SERS probes in a two-step sandwich immunoassay. As shown in Figure 2b, the lateral flow strip contains a streptavidin-coated test strip. Biotinylated secondary antibodies (Bio-Ab_2_) bind to the test strip to specifically capture the immune complex consisting of the SERS probe and the target antigen. In the presence of the MPXV antigen, a sandwich complex is formed, and the probe is immobilized on the test strip through strong biotin-streptavidin interactions. The system generates a strong Raman signal at 521 cm^−1^ corresponding to the IR808 reporter, enabling sensitive and specific detection of the target antigen. The two-step flow architecture enhances target binding and signal intensity while minimizing nonspecific accumulation. The total analysis time is less than 15 min, making it suitable for rapid point-of-care diagnostics.

Wang et al. [44] developed a SERS-LFA platform to detect three high-risk bacterial pathogens: *Yersinia pestis*, *Francisella tularensis*, and *Bacillus anthracis*. The growing threat of natural or intentional outbreaks, including high-risk bacterial pathogens such as *Y. pestis*, *F. tularensis*, and *B. anthracis*, requires rapid and sensitive diagnostic platforms for on-site biodefense [45]. Traditional culture-based methods are time-consuming and require centralized laboratory infrastructure, while molecular diagnostics are rapid but often rely on nucleic acid extraction, low temperature transport and storage systems, and skilled personnel [46,47]. Immunochromatographic assays offer portability and ease of use, but often lack the sensitivity and specificity needed for early detection of low-concentration bacterial targets [48]. To overcome these shortcomings, they used gold nanoparticles labeled with MGITC (malachite green isothiocyanate) as SERS probes, and constructed separate antibody-lined test strips for each bacterial target. As illustrated in Figure 2c, upon application of the bacterial sample, specific antigen–antibody interactions formed immunocomplexes that accumulated on the test lines. These complexes generated Raman signals that were quantitatively analyzed, allowing for highly sensitive detection. The platform employed individual lateral flow strips for each bacterial target, where MGITC-labeled gold nanoparticles were conjugated with monoclonal antibodies specific to *Y. pestis*, *F. tularensis*, or *B. anthracis*, respectively. Each strip featured a test line immobilized with the corresponding capture antibody, enabling pathogen-specific signal generation without cross-reactivity. Raman signals were measured at 1614 cm^−1^, characteristic of MGITC. The assay achieved detection limits of 43.4 CFU/mL for *Y. pestis*, 45.8 CFU/mL for *F. tularensis*, and 357 CFU/mL for *B. anthracis*, with a total assay time of under 15 min. These results demonstrate the potential of SERS-LFA technology for biothreat surveillance, emergency response, and early screening of exogenous bacterial pathogens in resource-limited or field environments.

Liang et al. [49] proposed a hybrid platform that integrates SERS and photothermal effects into a single LFA system to detect respiratory virus antigens, including influenza A (IAV), influenza B (IBV), and SARS-CoV-2 N protein, as a way to improve diagnostic accuracy and facilitate dual-mode signal detection. Respiratory viruses such as IAV, IBV, and SARS-CoV-2 are the leading causes of acute respiratory illness and often co-exist during seasonal epidemics and pandemics, complicating clinical diagnosis and response [50,51,52]. Early and accurate detection is essential for timely treatment, effective triage, and outbreak management. Nucleic acid amplification tests (NAATs), such as the commonly used RT-PCR, provide high sensitivity but are expensive, require complex instrumentation, and are not suitable for point-of-care diagnostics [53]. In contrast, antigen-based lateral flow tests offer portability and speed, but have low sensitivity and limited multiplexing, making them less effective at detecting multiple respiratory viruses simultaneously [54]. However, their platform utilized gold-core/silver-shell SERS nanotags (Au@Ag NPs) encoded with p-ATP (4-aminothiophenol) as multifunctional probes, generating both Raman and photothermal signals upon laser excitation. As shown in Figure 2d, this dual-modal design enabled complementary readouts using either a portable Raman spectrometer or a handheld thermal imager, thereby enhancing both analytical sensitivity and field utility. The assay achieved detection limits as low as 31.25 pg/mL for SARS-CoV-2 N protein and 15.63 pg/mL for ΔT-based photothermal detection (ΔT, defined as the temperature difference before and after laser excitation). Clinical validation involving 193 nasopharyngeal swab samples demonstrated >95% agreement with RT-PCR results, and importantly, the platform supported multiplex detection without signal cross-reactivity, addressing a major limitation of conventional LFA formats.

Collectively, these representative studies illustrate the versatility and clinical potential of SERS-LFA platforms in the diagnosis of infectious diseases caused by exogenous pathogens. The integration of advanced nanomaterials, multimodal detection strategies, and portable readout systems has led to substantial improvements in sensitivity, specificity, and operational speed in SERS-LFAs when compared to conventional LFA formats. Across exogenous targets, strip-based SERS lateral flow assays cluster into four method patterns: serology assays that measure virus-specific antibodies with reporter-encoded nanotags; antigen assays in sandwich format that deploy resonance dyes or gap engineered reporters for swab matrices; pathogen-specific designs that use separate test lines or parallel strips with monoclonal antibodies; and dual modal SERS/photothermal designs that yield complementary Raman and thermal readouts in field conditions.

### 3.2. SERS-LFA: Endogenous Diagnostic Case

**Figure 3:**
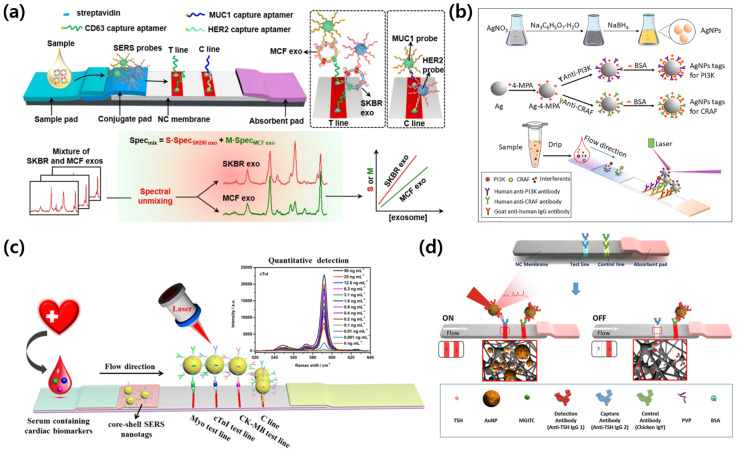
Representative SERS-integrated LFA platforms for endogenous disease diagnostics. (**a**) SERS-LFA strip for absolute quantification and classification of serum exosomes derived from breast cancer patients using spectral unmixing of multiplex Raman signals. Reprinted (adapted) with permission from ref [55]. Copyright 2023 ACS Publications. (**b**) SERS-LFA platform using AgNP tags for long-term monitoring of maternal blood biomarkers associated with intrauterine growth restriction. Reprinted (adapted) with permission from ref [56]. Copyright 2022 Elsevier. (**c**) Multiplex SERS-LFA using core–shell nanotags for the quantitative detection of cardiac biomarkers (Myo, cTnI, CK-MB) in serum. Reprinted (adapted) with permission from ref [60]. Copyright 2018 Elsevier. (**d**) Ultrasensitive SERS-based LFA for quantitative analysis of thyroid-stimulating hormone (TSH). Reprinted (adapted) with permission from ref [64]. Copyright 2017 Elsevier.

Shifting from exogenous pathogens to endogenous biomarkers, SERS-integrated lateral flow implementations addressing quantitative detection of low-abundance targets in oncology, obstetrics, cardiology, and endocrinology are surveyed. The case studies presented include exosome profiling, dual-protein prenatal monitoring, a three-marker acute myocardial infarction panel, and ultrasensitive hormone assays. All platforms are based on reporter-encoded nanotags with calibrated spectral readouts, and representative layouts are shown in Figure 3.

Su et al. [55] developed a SERS-LFA platform for the absolute quantification of serum exosomes derived from breast cancer patients, addressing the need for a minimally invasive yet highly sensitive diagnostic tool capable of molecular subtyping. The platform incorporated two distinct SERS probes encoded with DTNB (5,5′-dithiobis(2-nitrobenzoic acid)) and 4-MBA, functionalized with aptamers targeting human epidermal growth factor receptor 2 (HER2) and mucin-1 (MUC1), respectively. This dual-probe design enabled the selective recognition and spectral discrimination of exosomes secreted from HER2-positive (SKBR-3-derived) and luminal A (MCF-7-derived) breast cancer cells. Exosomes were captured on a test line by CD63 aptamers, and the resulting mixed SERS signals from the bound nanotags were processed using multivariate curve resolution-alternating least squares (MCR-ALS) to deconvolute the spectral contributions of HER2 and MUC1. As illustrated in Figure 3a, the test line accumulates a heterogeneous population of exosomes, whose molecular origins are resolved into their spectral fingerprints, allowing for accurate classification. The system achieved detection limits of 3.27 × 10^6^ particles/mL for SKBR exosomes and 4.80 × 10^6^ particles/mL for MCF exosomes in human serum. Importantly, the platform successfully distinguished between pre- and post-operative patient samples, underscoring its potential in molecular subtype classification and noninvasive therapeutic monitoring for breast cancer management. The same design principles extend from exosome profiling to soluble protein panels in strip-based SERS immunoassays. Reporter-encoded Au or Au/Ag nanotags in a sandwich format allow for quantitative readout using calibration curves with spectral deconvolution or ratiometric normalization, and blocking with optimized washing reduces serum-derived interference. Numerous multiplex implementations employ vertical flow geometries; representative results are summarized in Section 3.4 and shown in Figure 5c.

Song et al. [56] proposed a dual-signal surface-enhanced Raman-scattering lateral flow assay (SERS-LFA) for the simultaneous quantification of two critical protein biomarkers: phosphoinositide 3-kinase (PI3K) and serine/threonine-protein kinase CRAF (also known as RAF1). It is evident that both proteins play pivotal roles in placental and fetal growth signaling pathways and have been implicated in the pathogenesis of intrauterine growth restriction (IUGR). In the domain of maternal–fetal medicine, the early detection of IUGR is imperative for enhancing perinatal outcomes and facilitating timely intervention [57]. Impaired placental development and insufficient fetal nutrient supply are frequently associated with IUGR; however, current diagnostic methods, such as ultrasound measurements, lack molecular specificity and may fail to detect subtle or early-stage abnormalities [58]. Consequently, there is an urgent need for a rapid, sensitive, and biomarker-based platform that enables a quantitative assessment of molecular signals associated with fetal development [59]. The platform utilized silver nanoparticles (AgNPs) functionalized with 4-MPA (4-mercaptophenylboronic acid) as SERS tags, which were individually conjugated to anti-PI3K and anti-CRAF antibodies. These immunoprobes were incorporated into a lateral flow assay (LFA) strip containing two spatially separated test lines, each corresponding to one of the target biomarkers. This enabled multiplexed detection from a single maternal serum sample. As demonstrated in Figure 3b, the system schematic illuminates the synthesis of the SERS-tagged probes, the dual test-line layout on the strip, and the Raman spectroscopic detection mechanism, where the intensity of each signal directly correlates with the target protein concentration. The assay demonstrated exceptional analytical sensitivity, achieving detection limits of 0.76 fg/mL for PI3K and 0.61 fg/mL for CRAF. Importantly, the validation of the platform using clinical serum samples from 214 pregnant women revealed strong concordance with conventional enzyme-linked immunosorbent assay (ELISA) results, thus confirming the clinical feasibility of this platform for early diagnosis and longitudinal monitoring of IUGR in a non-invasive and rapid format.

Zhang et al. [60] developed a multiplexed SERS-LFA for the simultaneous detection of three cardiac biomarkers: myoglobin (Myo), cardiac troponin I (cTnI), and creatine kinase-MB (CK-MB). This innovation addresses the urgent need for rapid and accurate diagnosis of acute myocardial infarction (AMI) at the point of care. Early diagnosis of AMI critically depends on the timely and sensitive measurement of these biomarkers, which can vary significantly in concentration, ranging from sub-ng/mL to hundreds of ng/mL, and exhibit dynamic changes during the initial hours following cardiac injury [61,62]. Although conventional lateral flow assays are convenient, they often suffer from poor sensitivity and lack quantitative precision, particularly in multiplexed formats [63]. Consequently, there is a strong clinical demand for a platform that can detect multiple cardiac markers with high sensitivity and a wide dynamic range in a rapid and user-friendly manner. To address this challenge, the authors developed a surface-enhanced Raman-scattering lateral flow assay (SERS-LFA) utilizing silver–gold core–shell nanoparticles (Ag@Au NPs) embedded with NBA (Nile Blue A) as a Raman reporter. The nanoparticles were functionalized with detection antibodies specific to Myo, cTnI, and CK-MB, and were immobilized in the conjugate pad of the LFA strip. Capture antibodies were printed along three distinct test lines on a nitrocellulose membrane, each corresponding to one biomarker. As illustrated in Figure 3c, the multiplexed strip design facilitates the spatial separation of the immunoassay zones, and Raman signals specific to each marker were acquired using a 785 nm excitation laser, with the NBA peak monitored at 592 cm^−1^. The platform demonstrated exceptional analytical performance, achieving detection limits of 0.01 ng/mL for cTnI, 0.02 ng/mL for CK-MB, and 0.1 ng/mL for Myo, along with a broad dynamic range extending up to 500 ng/mL, encompassing clinically relevant concentrations. Validation with human serum samples revealed a strong correlation with results from conventional chemiluminescence immunoassay systems. These findings underscore the platform’s potential for bedside multiplexed cardiac event detection, enabling early risk stratification and rapid clinical decision-making in emergency settings.

Choi et al. [64] developed a SERS-LFA for the ultrasensitive detection of thyroid-stimulating hormone (TSH), addressing a critical diagnostic need for identifying abnormally low TSH concentrations associated with hyperthyroidism. TSH is a key endocrine biomarker secreted by the anterior pituitary gland, which regulates thyroid hormone production and systemic metabolism [65]. Clinically, an accurate measurement of TSH is essential for diagnosing both hypothyroidism and hyperthyroidism [66]. However, conventional lateral flow assays typically lack the sensitivity required to detect TSH levels below 0.5 μIU/mL, the threshold at which hyperthyroidism is often suspected [67]. To address these limitations, the authors developed a SERS-LFA system utilizing MGITC-labeled gold nanoparticles as SERS tags, which were conjugated with anti-TSH antibodies. These nanoparticles were integrated into a conventional LFA test strip architecture, which consists of a sample pad, conjugate pad, nitrocellulose membrane, and absorbent pad. As illustrated in Figure 3d, the immunoassay forms sandwich complexes on the test line upon the binding of TSH to the SERS-tagged detection antibodies. This interaction generates both a visible colorimetric signal and a Raman-active response. Raman mapping of the test line at 1615 cm^−1^ was conducted using a 633 nm excitation laser, facilitating quantitative signal acquisition. The assay demonstrated a detection limit of 0.025 μIU/mL, which is nearly two orders of magnitude lower than that of traditional colorimetric LFAs and well below the clinical threshold for hyperthyroidism detection. In addition to its high analytical sensitivity, the assay exhibited excellent reproducibility and completed measurements in under 10 min. These features underscore its potential as a rapid and reliable alternative to ELISA for routine monitoring of TSH levels in both clinical and decentralized testing environments. Collectively, these studies emphasize the expanding capabilities of SERS-LFA in diagnosing endogenous diseases with enhanced precision and speed. The integration of nanomaterials with tailored optical properties, advanced spectral deconvolution, and robust antibody/aptamer systems has resulted in platforms that offer unprecedented sensitivity, multiplexing, and field-applicability. As SERS instrumentation becomes increasingly portable and affordable, SERS-LFAs are poised to play a central role in future decentralized diagnostics and precision medicine applications [68].

These studies demonstrate that SERS-LFA technology has established itself as a useful tool for sensitive and efficient detection of endogenous biomarkers in various clinical fields such as cancer subtype classification, prenatal diagnosis, cardiovascular disease monitoring, and endocrine disease evaluation. Based on these technical achievements, recent studies have been conducted to improve the flow structure and secure signal uniformity. In the following section, we will examine major cases of vertical flow-based analysis platforms using SERS technology.

### 3.3. SERS-VFA: Exogenous Diagnostic Case

**Figure 4:**
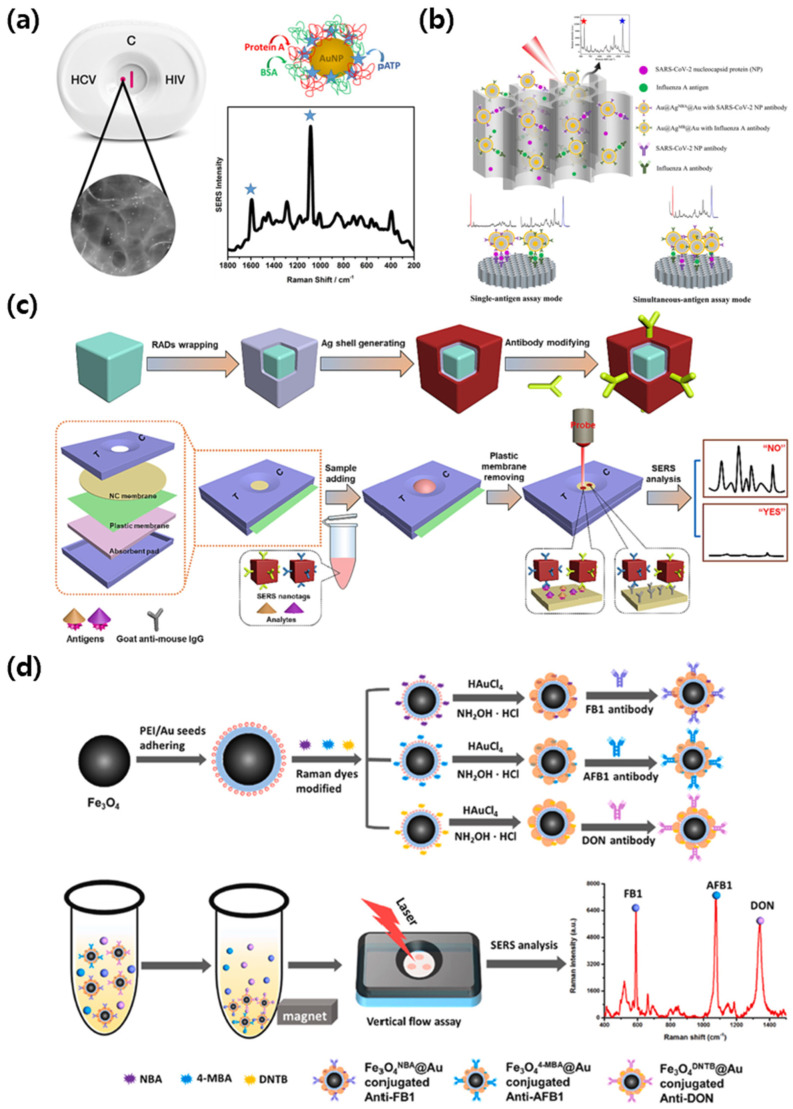
Representative SERS-integrated VFA platforms for exogenous disease diagnostics. (**a**) SERS-based VFA for multiplex detection of anti-HCV and anti-HIV antibodies using p-ATP-modified AuNPs as Raman probes. Reprinted (adapted) with permission from ref [69]. Copyright 2017 ACS Publications. (**b**) Dual-mode VFA platform for simultaneous detection of SARS-CoV-2 and Influenza A using Au@Ag@Au core–shell SERS nanotags and anodic aluminum oxide membranes. Reprinted (adapted) with permission from ref [72]. Copyright 2023 Royal Society of Chemistry. (**c**) Enhanced SERS-based VFA for multiplex detection of antibiotics using antibody-functionalized nanocube probes and Raman signal readout from membrane-immobilized capture zones. Reprinted (adapted) with permission from ref [76]. Copyright 2024 Elsevier. (**d**) Magnetic Fe_3_O_4_@Au SERS nanotag-based (VFA for the simultaneous detection of three foodborne mycotoxins (FB_1_, AFB_1_, and DON) using competitive immunoassay and multicolor Raman mapping. Reprinted (adapted) with permission from ref [80]. Copyright 2025 Elsevier.

Applying the same principles to the vertical flow format, SERS-integrated vertical flow assays use through thickness transport to shorten assay time, improve signal uniformity, and support compact multiplex layouts while preserving molecular sensitivity. For exogenous targets, representative method patterns include dot capture serology, dual antigen detection on anodic aluminum oxide membranes with core shell tags, and competitive assays for small molecules that use temporary flow restriction or magnetic enrichment. All platforms rely on reporter-encoded tags with calibrated spectral readouts, and representative implementations are shown in Figure 4.

Clarke et al. [69] developed a paper-based vertical flow assay integrated with SERS for the detection of anti- hepatitis C virus (HCV) antibodies. The early and accurate detection of blood-borne viral infections, such as human immunodeficiency virus (HIV) and HCV, remains critical for reducing disease transmission, initiating timely treatment, and improving long-term patient outcomes [70]. Rapid diagnostic tests based on LFA offer operational simplicity but frequently lack the sensitivity and quantitative capabilities needed for reliable antibody detection, particularly in asymptomatic or early-stage infections [71]. As illustrated in Figure 4a, the platform utilized 13 nm gold nanoparticles conjugated with protein A and labeled with p-ATP as a Raman reporter. The detection membrane was functionalized with preserved HCV viral antigens, enabling specific binding of anti-HCV antibodies in serum samples. The vertical flow configuration enabled rapid sample filtration and accumulation at the detection zone, forming a visible purple dot upon successful immunobinding. Raman measurements were performed using a 785 nm laser, and characteristic SERS peaks at 1083 cm^−1^ and 1590 cm^−1^ corresponding to p-ATP were used for detection. The platform achieved a visual limit of detection down to a 1/64 dilution of anti-HCV monoclonal antibody, corresponding to an antibody concentration of 53.1 µg/mL. The assay time was under 5 min, and the nitrocellulose membrane supported three-dimensional SERS signal acquisition with a coefficient variation of 16% across 10 different spots on the test zone. These findings demonstrate that SERS-enabled vertical flow formats can improve both the sensitivity and structural design of rapid antibody detection, with potential application in serological screening for infectious diseases in decentralized clinical environments.

Lu et al. [72] developed a dual-mode SERS-based vertical flow spectrometer that can simultaneously detect the nucleocapsid (NP) protein of SARS-CoV-2 and the hemagglutinin antigen of influenza A. When respiratory viruses such as SARS-CoV-2 and influenza A are prevalent at the same time, clinical symptoms may overlap, and accurate diagnosis is difficult because treatment protocols and public health responses for each virus are different [73,74]. Rapid and accurate differentiation of these pathogens is critical for appropriate patient management and infection control [75]. To address this challenge, this system used core–shell–core SERS nanotags composed of Au@Ag@Au, each encoded with a separate Raman reporter and functionalized with a virus-specific monoclonal antibody. These probes were designed to generate both Raman and photothermal signals under laser excitation, allowing cross-validation of the results through two independent detection modes. As shown in Figure 4b, the device, built on an anodic aluminum oxide (AAO) membrane to enhance vertical fluid flow and spatial resolution, enabled both single and simultaneous detection modes. In the simultaneous assay, SERS intensities at both 591 and 1621 cm^−1^ were quantitatively monitored without spectral overlap. The assay demonstrated detection limits of 0.47 pg/mL for SARS-CoV-2 NP and 0.62 pg/mL for influenza A antigen, with a linear range from 0.01 ng/mL to 1 µg/mL. In clinical evaluation, 20 throat swab samples were tested, including 3 SARS-CoV-2 positive and 17 negative samples. The method successfully distinguished between positive and negative groups with statistical significance (*p* < 0.01), and recoveries in spiked swabs ranged from 93.9 to 108.6% with coefficients of variation below 10%. These results indicate that the AAO-based SERS-VFA platform offers high sensitivity, specificity, and reproducibility for dual-virus detection, making it a promising tool for point-of-care respiratory virus diagnostics.

Liu et al. [76] developed a SERS-VFA using gold core–silver shell nanocubes as SERS nanotags for simultaneous detection of avermectins (AVM) and streptomycins (STR). The increasing presence of antibiotic residues in food poses a serious public health problem [77]. Veterinary antibiotic residues such as AVM and STR can accumulate through the food chain, causing nephrotoxicity and ototoxicity, and aggravating the problem of antibiotic resistance [78]. To ensure food safety, protocols that are sensitive, multiplexed, easily accessible, and operable without complex instrumentation or time-consuming are required [79]. The nanotags in this study were encoded with different Raman reporter molecules, DTNB for STR and 4-MBA for AVM, each of which was conjugated with corresponding monoclonal antibodies. A polyethylene terephthalate (PET) membrane was inserted above the nitrocellulose membrane to temporarily block vertical flow and increase the immunoreaction time, thereby enhancing signal intensity without complicating the assay operation. As illustrated in Figure 4c, the assay follows a competitive format in which free AVM and STR in the sample compete with immobilized antigens at the test dot, leading to reduced Raman intensity upon increasing analyte concentration. The platform achieved detection limits of 38.9 fg/mL for AVM and 29.1 fg/mL for STR, with linear dynamic ranges spanning from 1 pg/mL to 100 ng/mL for both antibiotics. In whole milk samples spiked with known concentrations, recoveries ranged from 93.3% to 109.1% for AVM and 93.5% to 111.7% for STR, with correlation coefficients of 0.9947 and 0.9932, respectively. The total assay time was less than 15 min, and results could be interpreted either by Raman readout or visually through color intensity at the test dot. These results demonstrate that the SERS-VFA system provides a powerful platform for rapid and ultrasensitive detection of antibiotic residues in complex food matrices and has substantial potential for on-site food safety monitoring.

Chen et al. [80] developed a VFA integrating SERS and magnetic enrichment for the simultaneous detection of fumonisin B1 (FB_1_), aflatoxin B1 (AFB_1_), and deoxynivalenol (DON). Accurately and simultaneously detecting multiple mycotoxins in agricultural products is imperative for ensuring food safety and public health [81]. Contaminants such as FB_1_, AFB_1_, and DON are commonly found in cereal grains and are known for their severe toxic effects, including hepatotoxicity, immunosuppression, and carcinogenicity [82]. These mycotoxins frequently co-occur in food and feed, necessitating a detection strategy that can efficiently identify multiple targets within a single assay [83,84]. There is an urgent requirement for the development of rapid, sensitive, and field-deployable methods to meet the growing demand for the routine screening of these hazardous compounds in complex food matrices [85,86]. To address this need, this platform utilizes Fe_3_O_4_@Au core–shell magnetic nanoparticles, each encoded with a unique Raman reporter molecule and conjugated with a monoclonal antibody that is specific for the target mycotoxin. The magnetic property of the probes enables efficient separation from complex grain extracts prior to assay loading, thereby minimizing matrix interference and improving signal clarity. As demonstrated in Figure 4d, the vertical flow apparatus incorporates three distinct test zones on a nitrocellulose membrane, each of which is pre-coated with a specific mycotoxin–BSA conjugate. The competitive immunoassay format utilized involves the competition between free mycotoxins present in the sample and immobilized antigens for binding to the antibody-labeled SERS nanotags. The Raman spectra acquired at 593, 1076, and 1335 cm^−1^ correspond to FB_1_, AFB_1_, and DON, respectively, enabling multiplexed signal differentiation. The assay achieved detection limits of 0.053 pg/mL for FB_1_, 0.028 pg/mL for AFB_1_, and 0.079 pg/mL for DON, with a broad linear dynamic range from 0.01 to 100 pg/mL. In wheat samples spiked with these mycotoxins, recoveries ranged from 86.6 to 108.4%, and correlation with ELISA yielded R^2^ values of 0.995, 0.985, and 0.981, respectively. The biosensor also demonstrated excellent selectivity, showing no cross-reactivity among the three targets or with structurally similar interferents such as FB_2_, AFB_2_, and NIV. The total assay time was under 15 min. These findings establish the proposed Fe_3_O_4_@Au SERS-VFA platform as a highly sensitive and practical tool for multiplex mycotoxin detection in complex agricultural samples.

These case studies comprehensively demonstrate the versatility and analytical power of SERS-integrated VFA for the detection of exogenous disease targets. By combining the high sensitivity and molecular specificity of SERS with the rapid fluid transport and structural efficiency of VFA, these platforms can address several limitations of conventional lateral flow diagnostics. Across exogenous targets, vertical flow SERS implementations for virus, bacterial, and toxin detection cluster into several method patterns. Dot capture antibody assays exploit rapid filtration on the capture membrane, with protein A conjugated Au nanoparticles and reporter-encoded tags for calibrated Raman readout from serum. Dual antigen configurations deploy core shell Au@Ag@Au nanotags on anodic aluminum oxide membranes to promote uniform vertical transport and clear spectral separation for simultaneous measurement. For small molecules and toxins, competitive formats use immobilized antigen conjugates and improve reaction efficiency by inserting a thin PET layer to temporarily slow flow or by using Fe_3_O_4_@Au magnetic tags for preconcentration. These layouts retain microliter inputs and minute scale analysis times and can be paired with photothermal readout for confirmation. Applications ranging from viral antigen identification to antibiotic residue monitoring demonstrate the adaptability of SERS-VFA in clinical, environmental, and food safety settings. In addition to infectious agents and environmental contaminants, recent studies have begun to explore the use of SERS-VFA for the detection of endogenous biomarkers associated with chronic and non-communicable diseases.

### 3.4. SERS-VFA: Endogenous Diagnostic Case

**Figure 5:**
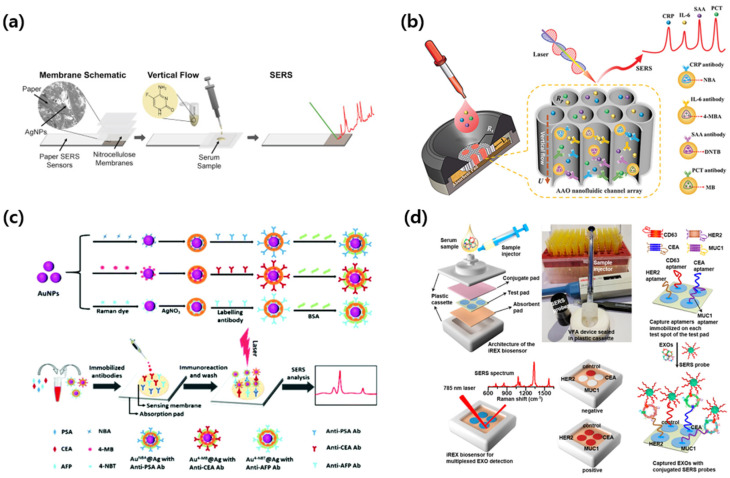
Representative SERS-integrated VFA platforms for endogenous disease diagnostics. (**a**) Paper-based vertical flow SERS assay for therapeutic drug monitoring of flucytosine in serum using inkjet-printed AgNP substrates and nitrocellulose membrane-assisted separation. Reprinted (adapted) with permission from ref [87]. Copyright 2017 Elsevier. (**b**) Multiplex detection of inflammatory biomarkers (CRP, IL-6, SAA, PCT) using SERS-labeled antibodies and an AAO nanochannel vertical flow array. Reprinted (adapted) with permission from ref [91]. Copyright 2020 Wiley-VCH. (**c**) Vertical flow SERS immunoassay for simultaneous detection of PSA, CEA, and AFP using AuNP-based Raman nanotags. Reprinted (adapted) with permission from ref [99]. Copyright 2019 Royal Society of Chemistry. (**d**) iREX vertical flow system for exosome-based multiplex detection of HER2, CEA, and MUC1 using aptamer-modified capture zones and SERS-tagged AuNPs. Reprinted (adapted) with permission from ref [105]. Copyright 2023 ACS Publications.

Endogenous biomarkers measured by SERS-integrated vertical flow assays are considered, including therapeutic drug monitoring, inflammation panels, protein tumor markers, and exosome-based liquid biopsy. Emphasis is placed on quantitative multi analyte readouts from microliter inputs with minimal preprocessing and minute-scale analysis times, with good agreement to laboratory immunoassays. Representative designs, reporters, matrices, and performance are summarized in Figure 5 and are detailed in the case studies that follow.

Berger et al. [87] developed a paper-based vertical flow SERS assay for the detection of flucytosine in undiluted serum samples. Therapeutic drug monitoring (TDM) plays a critical role in optimizing therapeutic efficacy and minimizing toxicity, especially for drugs with narrow therapeutic ranges [88]. Flucytosine, a commonly used antifungal agent for the treatment of cryptococcal meningitis, requires precise dose adjustment due to its potential for nephrotoxicity and bone marrow suppression [89]. Conventional TDM methods, such as high-performance liquid chromatography or mass spectrometry, offer high sensitivity, but their complexity and infrastructure requirements often make them impractical for near-patient testing [90]. Offering a more accessible alternative, the platform consists of a three-layer nitrocellulose membrane and a printed silver nanoparticle SERS substrate fabricated by inkjet printing. This configuration enables vertical sample filtration and target accumulation, effectively removing large proteins and matrix contaminants without prior sample preparation. As shown in Figure 5a, flucytosine interacts with the AgNP region and produces a strong SERS signal with a characteristic peak at 784 cm^−1^, corresponding to its ring-breathing mode. The assay demonstrated quantitative detection over the full therapeutic range of flucytosine (10–100 µg/mL), with a limit of detection down to 10 µg/mL in undiluted serum. The full analysis could be completed in approximately 15 min. The system showed good linearity (R^2^ = 0.971) and reproducibility across multiple sensors, confirming its potential as a portable and cost-effective platform for near-patient therapeutic drug monitoring.

Chen et al. [91] developed a vertical-flow SERS biosensor based on a nanofluidic channel array fabricated with anodized aluminum oxide (AAO) membranes. Systemic inflammation is a key response in the pathophysiology of many acute and chronic diseases [92]. Biomarkers such as C-reactive protein (CRP), interleukin-6 (IL-6), serum amyloid A (SAA), and procalcitonin (PCT) are widely used for diagnosis and treatment-monitoring of various diseases, ranging from infection and sepsis to cardiovascular and autoimmune diseases [93,94,95]. However, simultaneous detection of these biomarkers typically requires multi-step assays performed in central laboratories and is challenging for rapid, multiplexed inflammation profiling at the point of care [96,97,98]. To overcome these challenges, this system uses gold-core–silver-shell SERS nanotags encoded with four Raman reporter molecules conjugated with antibodies targeting CRP, IL-6, SAA, and PCT. The Raman reporters used were NBA for CRP (593 cm^−1^), 4-MBA for IL-6 (1075 cm^−1^), DTNB for SAA (1341 cm^−1^), and methylene blue for PCT (1621 cm^−1^). These nanotags were premixed with serum samples and introduced into the AAO-based VFA, which contained spatially separated capture zones functionalized with specific antibodies. As shown in Figure 5b, the vertically aligned nanochannels facilitated rapid and uniform fluid transport, promoting efficient immunoreactions and minimizing cross-reactivity. The multiplexed Raman signals were clearly distinguishable and could be quantitatively analyzed using spectral deconvolution. The assay achieved detection limits of 0.32 ng/mL for CRP, 0.30 ng/mL for IL-6, 0.37 ng/mL for SAA, and 0.35 ng/mL for PCT. In a clinical validation study using spiked human serum samples, the assay demonstrated excellent agreement with ELISA, with R^2^ values of 0.989 for CRP, 0.996 for IL-6, 0.996 for SAA, and 0.983 for PCT. Recovery rates ranged from 88.7% to 106.4%, and the total assay time was less than 10 min without requiring sample pretreatment. These results confirm that the AAO-based SERS-VFA platform offers a rapid, sensitive, and practical solution for multiplex inflammation profiling in clinical diagnostics.

Chen et al. [99] developed a vertical flow SERS immunoassay for the multiplexed detection of prostate-specific antigen (PSA), carcinoembryonic antigen (CEA), and alpha-fetoprotein (AFP) using a single compact detection area. Early detection of cancer can significantly improve treatment outcomes, and simultaneous measurement of multiple tumor biomarkers can significantly improve diagnostic accuracy [100]. Protein biomarkers such as PSA, CEA, and AFP are widely used for the diagnosis and monitoring of prostate, gastrointestinal, and liver cancers [101,102,103]. However, existing simultaneous diagnostic methods are generally labor-intensive and rely on complex equipment, making them unsuitable for rapid testing or POC [104]. To address these limitations, this platform was designed to enable high sensitivity and simultaneous quantification of multiple protein biomarkers in a portable format. The system utilized gold-core–silver-shell SERS nanotags encoded with three Raman reporters. PSA was characterized by a specific signal at 593 cm^−1^ using NBA as a Raman reporter, CEA by 4-MBA at 1074 cm^−1^, and AFP by 4-nitrothiophenol at 1343 cm^−1^, respectively. The nanotags were conjugated with monoclonal antibodies that were specific for each biomarker and premixed with serum samples. All three capture antibodies were immobilized onto a single circular test zone on a nitrocellulose membrane. As shown in Figure 5c, the Raman signals of all three biomarkers were simultaneously captured and quantitatively analyzed using spectral deconvolution. The method achieved detection limits of 0.37 pg/mL for PSA, 0.43 pg/mL for CEA, and 0.26 pg/mL for AFP. The linear range was from 1 pg/mL to 10 μg/mL for PSA, and from 1 pg/mL to 1 μg/mL for both CEA and AFP. Clinical validation results using five human serum samples showed a strong correlation with ELISA, with R-squared values greater than 0.98 for all three targets. The recoveries ranged from 90.1% to 111.4%, and the coefficient of variation was less than 13%. Taken together, multiplex vertical flow assays provide a practical route to rapid cancer biomarker profiling. Small sample volumes and short assay times are preserved, while measurements acquired in one detection zone allow for straightforward normalization across markers and robust comparison with ELISA. These results demonstrate that the vertical flow SERS method provides a practical and reliable platform for rapid and multiplexed cancer biomarker analysis and holds promise for PoC cancer diagnosis.

Su et al. [105] developed an integrated Raman exosome biosensor (iREX) for simultaneous analysis of epidermal growth factor receptor 2 (HER2), carcinoembryonic antigen (CEA), and mucin 1 (MUC1) proteins on the surface of circulating exosomes in serum samples using a vertical flow SERS platform. Accurate molecular subtype analysis of cancer is essential to guide targeted therapy and improve patient prognosis [106]. Conventional tissue biopsies are invasive, have limited sampling frequency, and often fail to capture tumor heterogeneity [107]. Exosomes, nano-sized extracellular vesicles secreted by tumor cells, contain surface proteins that reflect the molecular characteristics of their origin [108]. Found in blood and other body fluids, exosomes are attractive targets for noninvasive diagnostics, but their small size and low abundance require highly sensitive and multiplexed detection strategies [109]. To address these clinical challenges, the device consists of a plastic cassette containing a multilayer vertical flow pad with three spatially separated test sites, each immobilized with a specific capture aptamer targeting one of the exosome markers. The SERS nanotags are encoded with separate Raman reporters and conjugated to a CD63 aptamer, which is designed to selectively bind to the exosome surface. As shown in Figure 5d, the vertical flow configuration allowed for rapid passage of serum samples and efficient capture of exosome-SERS probe complexes in the designated test zone. Raman peaks corresponding to NBA (HER2, 593 cm^−1^), 4-MBA (CEA, 1075 cm^−1^), and DTNB (MUC1, 1331 cm^−1^) were clearly distinguished and used for quantitative analysis. The analysis showed limits of detection of 6.25 × 10^5^ particles per microliter for HER2-expressing exosomes, 1.25 × 10^5^ particles per microliter for CEA, and 2.5 × 10^4^ particles per microliter for MUC1. The system was able to distinguish HER2-positive, CEA-positive, and triple-negative exosome samples, showing strong agreement with conventional exosome characterization methods. Importantly, the entire assay was completed in less than 10 min, and the signal intensities showed a linear response of more than two orders of magnitude for each marker. These results demonstrate the potential of the SERS-integrated vertical flow platform to perform rapid multiplexed liquid biopsy assays based on exosome profiling, enabling minimally invasive cancer diagnosis and real-time molecular monitoring.

To synthesize these advances and enable a side-by-side comparison across exogenous and endogenous applications in both LFA and VFA, Table 1 consolidates representative performance metrics, including assay format, reporter choice, limit of detection, sample matrix, analysis time, and multiplexing.

These case studies comprehensively demonstrate the expanded capabilities of SERS-integrated vertical flow spectrometry for endogenous biomarker detection across a range of clinical applications. From therapeutic drug monitoring and inflammation profiling to multiplexed cancer biomarker detection and exosome-based liquid biopsies, SERS-VFA has demonstrated exceptional sensitivity, specificity, and multiplexing performance within rapid analysis times and minimal sample requirements. Importantly, these platforms support minimally invasive diagnostics using small sample volumes, without the need for complex preprocessing steps or bulky equipment. As the demand for precision diagnostics continues to grow, particularly in the areas of cancer management and chronic disease monitoring, SERS-VFA is likely to play a key role in expanding access to high-performance molecular diagnostics in both clinical and resource-constrained settings.

## 4. Technical Challenges and Future Prospects

Despite promising research results of SERS-integrated flow-based assays, several technical challenges need to be addressed for widespread clinical applications. One of the major limitations is the reproducibility of SERS signals, which can be affected by inconsistencies in nanoparticle synthesis, aggregation, and non-uniform distribution across the assay membrane. In addition, background interference from complex biological matrices, such as whole blood or crude serum, can affect spectral clarity and quantitative accuracy. Although vertical flow approaches provide improved results compared to conventional lateral structures, problems such as non-uniform flow distribution and limited multiplexing capacity in a single assay area remain. Meanwhile, establishing a standardized protocol is necessary to improve the reproducibility and clinical applicability of SERS-based diagnostics. Standardization includes specifying operational parameters such as sample collection and storage conditions, process specifications for nanotag synthesis and surface chemistry, membrane pretreatment and blocking conditions, injection volume, and reaction time. Spectroscopic acquisition should consistently set and report laser wavelength and power, integration time, spot size, and mapping interval. Data processing should ideally include a preprocessing pipeline including cosmic ray removal, baseline correction, and normalization, preferably provided as an open, version-controlled code. Furthermore, cross-platform calibration using reference materials and representative reporter panels, membrane phase quality control using intra- and inter-line coefficient of variation criteria, and lot release testing and acceptance criteria for nanotags and membranes are required. To address these limitations, future research should focus on developing highly uniform and stable SERS substrates with tunable surface plasmon properties. Core–shell nanostructures offer important directions, including designing interparticle spacing, magnetic enrichment for selective target capture, and surface modification to minimize non-specific binding. On the structural side, microfluidic integration, 3D membrane stacking, and spatial encoding strategies can improve analyte separation and signal resolution in multi-analyte environments. In addition, computational tools such as multivariate spectral analysis and machine learning algorithms can improve signal interpretation, especially in complex or multi-analyte environments. Cloud-based data platforms and smartphone connectivity can facilitate real-time diagnostic decision-making and remote monitoring. From a clinical standpoint, future studies should aim to validate SERS-VFA performance in large-scale, multi-center clinical trials across diverse patient populations. Regulatory considerations, cost-effectiveness, and compatibility with existing diagnostic workflows will be key factors in transitioning these platforms from laboratory prototypes to commercial products. Taken together, these efforts will help advance SERS-integrated flow spectrometry into a robust, accessible, and intelligent diagnostic tool suitable for a wide range of clinical, environmental, and public health applications.

## 5. Conclusions

SERS-integrated flow-based analytical platforms have emerged as powerful tools to address the limitations of conventional lateral and vertical flow diagnostics. Combining the high sensitivity and molecular specificity of SERS with the operational simplicity and portability of flow-based approaches, SERS-LFA and SERS-VFA systems enable rapid, multiplexed, and quantitative detection of a wide range of disease-related biomarkers. Recent developments have demonstrated applicability for both exogenous and endogenous diagnostics, including detection of viral antigens, bacterial toxins, therapeutic drug concentrations, inflammatory markers, cancer-related proteins, and exosome-derived targets. The SERS-LFA platform offers a simple device architecture and is suitable for PoC applications, while the SERS-VFA system offers improved fluid dynamics and more uniform signal distribution, enabling higher throughput and improved analytical performance. Through the integration of core–shell SERS nanotags, competitive or sandwich immunoassays, and spectral deconvolution techniques, detection limits as low as pg per mL have been achieved, with high agreement with conventional laboratory assays such as ELISA and electrochemiluminescence immunoassays. As research in nanoprobe engineering, membrane design, and portable detection devices continues to advance, SERS-based flow diagnostics are expected to expand their roles in clinical decision-making, early disease detection, and real-time treatment monitoring. Looking ahead, further progress will hinge on three priorities: standardization of operating and reporting protocols with reference materials and minimum reporting items to ensure inter-laboratory comparability; device and materials engineering that improve flow uniformity and lot-to-lot stability of reporter-encoded nanotags while maintaining antifouling surfaces and optional magnetic or dual-modal capabilities; and calibration transfer across devices, and compact readers with on-device analytics. Together, these steps will accelerate the transition of SERS-based flow diagnostics from robust prototypes to reliable tools for routine screening, triage, and longitudinal monitoring.

## Figures and Tables

**Table 1:** Consolidated comparison of representative SERS-integrated LFA and VFA.

Format	LFA	LFA	LFA	LFA	VFA	VFA	VFA	VFA
**Target type**	Exogenous	Exogenous	Endogenous	Endogenous	Exogenous	Exogenous	Endogenous	Endogenous
**Analyte(s)**	SARS-CoV-2 IgG	IAV, IBV, SARS-CoV-2 N	Myoglobin, cTnI, CK-MB	TSH	SARS-CoV-2 NP, Influenza A HA	FB1, AFB1, DON	CRP, IL-6, SAA, PCT	Exosomal HER2, CEA, MUC1
**Probe or reporter**	Ag@Au, 4-MBA	Au@Ag, p-ATP dual modal	AuNP, MGITC	Au@Ag@Au	Fe_3_O_4_@Au, NBA; 4-MBA; etc.	Au@Ag tags, NBA; 4-MBA; DTNB; MB	Au@Ag tags, NBA; 4-MBA; DTNB; MB	AuNP tags, NBA; 4-MBA; DTNB
**LoD**	0.52 pg mL^−1^	31.25 pg mL^−1^ SERS; 15.63 pg mL^−1^ ΔT	0.10; 0.01; 0.02 ng mL^−1^	0.025 μIU mL^−1^	0.47; 0.62 pg mL^−1^	0.053; 0.028; 0.079 pg mL^−1^	0.32; 0.30; 0.37; 0.35 ng mL^−1^	6.25 × 10^5^; 1.25 × 10^5^; 2.5 × 10^4^ particles μL^−1^
**Matrix**	Serum	Nasopharyngeal swab	Serum	Not specified	Throat swab	Wheat extract	Serum	Serum
**Assay time**	Not specified	Not specified	Not specified	Less than 10 min	Not specified	Less than 10 min	Less than 10 min	Less than 10 min
**Multiplex**	No	3-plex	3-plex	No	2-plex	3-plex	4-plex	3-plex
**Ref.**	[37]	[49]	[61]	[65]	[75]	[83]	[70]	[107]

## Data Availability

No new data were created in this work.
